# A Pathogenic L2HGDH Variant Impairs Mitochondrial Targeting and Enzyme Function in L-2-Hydroxyglutaric Aciduria: Clinical and Functional Evidence from Two Affected Siblings

**DOI:** 10.3390/genes16080982

**Published:** 2025-08-20

**Authors:** Qiang Guo, Thilo Löhr, Patrick Giavalisco, Vera Riehmer, Hans Zempel

**Affiliations:** 1 Department of Clinical Laboratory, Anhui Children’s Hospital, Hefei 230032, China; qiang.guo@uk-koeln.de; 2 Institute of Human Genetics, Faculty of Medicine, University Hospital Cologne, University of Cologne, 50931 Cologne, Germany; 3 Center for Molecular Medicine Cologne (CMMC), University of Cologne, 50931 Cologne, Germany; 4 Metabolomics Core Facility, Max Planck Institute for Biology of Ageing, 50931 Cologne, Germany

**Keywords:** L-2-hydroxyglutaric aciduria 2, L-2-hydroxyglutarate dehydrogenase 3, metabolic disorder disease

## Abstract

Background: L-2-hydroxyglutaric aciduria (L2HGA) is a rare autosomal recessive neurometabolic disorder caused by biallelic loss-of-function variants in the L-2-hydroxyglutarate dehydrogenase (*L2HGDH*) gene, leading to accumulation of L-2-hydroxyglutarate in the brain and other tissues. While various variants have been reported, the pathogenic mechanism of specific variants remains unclear. In this study, we aimed to investigate the molecular consequences of the c.905C>T p.(Pro302Leu) variant, identified in two siblings with typical symptoms of L2HGA, by analyzing its effects on protein localization and enzymatic activity in a cell model. Methods: HA-tagged wild-type and p.(Pro302Leu) mutant *L2HGDH* constructs were overexpressed in HEK293T cells. We assessed protein expression, subcellular localization, and enzymatic activity using Western blot analysis, immunofluorescence microscopy, and a specific enzyme assay measuring 2,6-dichloroindophenol (DCIP) reduction to assess L2HGDH enzymatic activity. Results: Western blotting showed that wild-type *L2HGDH* exists primarily in the processed, mature mitochondrial form, whereas the p.(Pro302Leu) mutant remained largely in the unprocessed precursor form. Immunofluorescence and differential centrifugation revealed that wild-type protein localized to mitochondria, while the mutant protein accumulated in the cytoplasm in a diffuse or punctate pattern. Enzyme activity assays demonstrated that the mutant retained <30% of wild-type activity. Conclusions: These findings indicate that the p.(Pro302Leu) variant leads to aggregation of mislocalized protein, thereby impairing L2HGDH function rather than decreasing enzymatic function. This study provides clinical and molecular evidence supporting the pathogenicity of this previously reported mutation and highlights the importance of mitochondrial import for enzyme functionality in L2HGA.

## 1. Introduction

L-2-hydroxyglutaric aciduria (OMIM #236792) is a rare metabolic disorder accompanied by increased levels of L-2-hydroxyglutarate in bodily fluids and L-2-hydroxyglutarate accumulation in several organs, i.e., the brain, resulting in neurological impairment. First identified in 1980 [1], there have been fewer than 500 (Leiden Open Variation Database) documented cases globally. Orphanet classifies the disease as having a prevalence of less than 1 in 1,000,000, (Orphanet ORPHA: 79314). The condition follows an autosomal recessive pattern of inheritance, with the *L2HGDH* gene responsible for producing the enzyme L-2-hydroxyglutarate dehydrogenase. Deficiencies in this enzyme arise from variants in *L2HGDH*, disrupting the function of L2HGDH, the typical breakdown of L-2-hydroxyglutarate into α-ketoglutarate [2]. As a result, L-2-hydroxyglutarate builds up to excessive levels in the plasma, urine, and cerebrospinal fluid of affected individuals [3], which is believed to have neurotoxic effects that can lead to brain damage.

Clinically, L2HGA usually manifests in early childhood through developmental delays or regressions, seizures, cerebellar ataxia, and extrapyramidal symptoms. Intellectual disability typically becomes evident, and many patients experience macrocephaly and speech challenges [4]. Brain magnetic resonance imaging (MRI) shows distinct abnormalities, particularly bilateral and symmetric subcortical white matter hyperintensities, often affecting the basal ganglia and dentate nuclei [5,6]. These neuroimaging characteristics are highly indicative of L2HGA when considered alongside the clinical context. Elevated levels of L-2-hydroxyglutarate, as confirmed by organic acid analysis, support the biochemical diagnosis [2].

The *L2HGDH* gene was identified in 2004 through studies that mapped L2HGA to chromosome 14q22.1 and demonstrated that variants in *L2HGDH* abrogate L-2-hydroxyglutarate dehydrogenase activity [7]. L2HGDH is a nuclear-encoded, FAD-dependent enzyme that localizes to mitochondria and catalyzes the oxidation of L-2-hydroxyglutarate to α-ketoglutarate [7]. Pathogenic variants in *L2HGDH* generally result in loss of enzyme function and failure to metabolize L-2-hydroxyglutarate. As of May 2025, the Leiden Open Variation Database (LOVD) has reported 111 variants associated with *L2HGDH* deficiency. These include missense substitutions like p.(Gly60Arg), as well as frameshift variants (c.180delG) [8], or nonsense variants (c.751C>T) that produce a truncated, nonfunctional enzyme. Despite the variety of variants, nearly all characterized cases show a common outcome of accumulation of toxic metabolite.

Here, we describe two siblings from a consanguineous family diagnosed with L2HGA, in whom we identified a homozygous missense variant in *L2HGDH*: c.905C>T, resulting in a p.(Pro302Leu) substitution. We performed a series of molecular experiments to characterize the effect of this variant on the L2HGDH protein. In particular, we examined whether the p.(Pro302Leu) change affects the enzyme’s expression, mitochondrial localization, and catalytic activity. Our results provide insight into the pathogenic mechanism of this variant and expand the understanding of genotype–phenotype correlations in L2HGA.

## 2. Materials and Methods

### 2.1. Sanger Sequencing for Variant Confirmation

The variant was previously identified using next-generation sequencing (NGS) for the older brother, and it was confirmed here by Sanger sequencing in the younger brother and parents. For this, genomic DNA was extracted from peripheral blood samples collected from the two affected siblings and their parents using standard extraction procedures. Approximately 3–5 mL of whole blood was collected in EDTA-containing tubes and processed within 24 h. The L2HGDH (transcript: ENST00000267436.8) gene was amplified by polymerase chain reaction (PCR) using primers flanking exon 7, where the c.905C>T p.(Pro302Leu) variant is located (forward primer: GCTGCCACCTAAATGCTAAAG; reverse primer: AACCACAAGAGAAACCGATG). PCR products were purified using the ExoSAP-IT™ PCR Product Cleanup reagent (Thermo Fisher Scientific, Waltham, MA, USA) and subjected to Sanger sequencing using the ABI 3500 Genetic Analyzer (Applied Biosystems, Foster City, CA, USA). Sanger sequences were analyzed using JSI SequencePilot software 4.2 (JSI medical systems, Ettenheim, Germany) and aligned to the reference genome (GRCh37/hg19) to detect variants.

### 2.2. Measurement of L-2-Hydroxyglutarate Levels

Plasma samples (50 µL each) from two heterozygous parents, two affected children and unrelated healthy controls were analyzed for L-2-hydroxyglutarate levels. Metabolite extraction and measurements were performed using anion chromatography coupled with high resolution mass spectrometry (AIC-HR-MS) [9]. A commercial L-2-hydroxyglutarate standard (Sigma Aldrich, Taufkirchen, Germany) was used to confirm the identity of the AIC-HR-MS detected compound from serum samples. In the analyzed serum samples, two distinct peaks were detected at 3.21 min and 3.50 min, corresponding putatively to L and D-2-hydroxyglutarate, respectively.

### 2.3. Mitochondrial Isolation

Mitochondria were isolated from HEK293 cells 48 h post-transfection. Cells were collected on ice by gentle scraping and pelleted by centrifugation at 2500× *g* for 3 min at 4 °C. The cell pellet was resuspended in ice-cold mitochondrial isolation buffer (MIB), consisting of 6.846 g sucrose, 0.121 g Tris (100 mM), and 1 mL of 0.1 M EGTA dissolved in 80 mL of distilled water, with the pH adjusted to 7.4. The cell suspension was transferred to a pre-chilled Dounce homogenizer and homogenized with 25 strokes on ice. The homogenate was centrifuged at 600× *g* for 10 min at 4 °C to remove unbroken cells and nuclei. The supernatant was carefully collected and further centrifuged at 10,000× *g* for 10 min at 4 °C. The resulting pellet, containing crude mitochondria, was resuspended in appropriate buffer and used for downstream analyses.

### 2.4. Cloning of L2HGDH and Mutated L2HGDH

The coding sequence for human *L2HGDH* (kindly provided by Prof. Dr. Sunil Sudarshan, Department of Urology, University of Birmingham, Alabama) was cloned into a pAAV expression vector (Addgene #59462, Cambridge, MA, USA). For this, *L2HGDH* was PCR-amplified, flanked with restriction sites for BamHI (5′) and EcoRI (3′) and C-terminally tagged with an HA-tag. Fragments were loaded onto a 1% agarose gel and gel electrophoresis was conducted. The desired bands were cut out and purified using the NucleoSpin™ Gel & PCR Clean up Kit (Macherey & Nagel, Düren, Germany) according to the manufacturer’s protocol. DNA concentration was measured spectrophotometrically (NanoDrop™, Thermo Fisher Scientific, Waltham, MA, USA) and purified *L2HGDH* was cloned into a pAAV subvector using the CloneJET PCR Cloning Kit (TFS). For plasmid amplification, pAAV_*L2HGDH* was transformed into chemocompetent bacteria (*Escherichia coli* Top10™, Waltham, MA, USA) which were cultivated for 16–18 h at 37 °C on agar plates with antibiotics. Colonies were picked and grown in nutrient medium containing antibiotics and plasmids were isolated and purified for sequencing and propagation using the PureYield™ Plasmid Miniprep and Midiprep kit (ProMega, Madison, WI, USA).

To introduce the disease-associated point variant c.905C>T, pAAV_L2HGDH was PCR-amplified using a primer pair that binds to the variant site and carries the variant. Application of the Q5^®^ Site-Directed Mutagenesis kit (New England Biolabs, Ipswich, MA, USA) according to the manufacturer’s protocol yielded a pAAV_mL2HGDH plasmid that was amplified as described above.

### 2.5. Transfection of HEK 293T Cells

HEK 293T cells were cultivated in DMEM plus GlutaMAX™ medium (Gibco, Waltham, MA, USA) with 10% FBS at 37 °C and 5% CO_2_ and maintained weekly. For transfection, HEK cells were either seeded for Western blot in 6-well plates with 300,000 cells/well in 2 mL medium, for immunostainings in 24-well plates on poly-d-lysine (PDL)-coated coverslips with 50,000 cells/well in 0.5 mL medium, or for enzyme activity assay in 10 cm dishes with 2,000,000 cells/dish in 10 mL medium. A total of 500–2000 ng of plasmid was mixed with 3 µL of polyethylenimine per 1 µg of DNA and adjusted to 200 µL with OptiMEM (Gibco) for Western blot, to 50 µL for immunostainings, and to 1 mL for the activity assay. The transfection mixture was incubated at room temperature (RT) for 20 min and added dropwise to the cells. Cells were incubated at 37 °C and 5% CO_2_ overnight.

### 2.6. Western Blot

Western blot was conducted to validate protein overexpression and thus plasmid functionality. Transfected cells were harvested in ice cold PBS, centrifuged, and the pellet was resuspended in RIPA cell lysis buffer supplemented with phosphatase and protease inhibitor. The mixture was incubated for 20 min at 4 °C with shaking at 600 rpm, then centrifuged for 20 min at 4 °C and 16,000× *g*. The supernatant was collected and used for a BCA protein assay applying the Pierce™ BCA Protein Assay Kit (TFS) according to the manufacturer’s protocol and measuring using a Safire2 microplate reader (Tecan, Männedorf, Switzerland). SDS-Page was conducted in a 10% acrylamide gel containing 0.5% (*v*/*v*) trichlorethanol (TCE) for normalization against total protein using a Mini-PROTEAN Tetra Cell Kit (Bio-Rad, Hercules, CA, USA). A total of 10 µg of protein samples were diluted with 3× Laemmli buffer, boiled at 95 °C for 5 min and the gel was run at 80 V for 20 min and another subsequent 100 min at 120 V. Total protein was visualized by exposure to UV light for 1 min and transferred on a polyvinylidene fluoride (PVDF) membrane using the Trans-Blot Turbo Transfer System (Bio-Rad). The membrane was blocked with 5% milk in TBS-T at RT for 1 h and incubated with α-L2HGDH primary antibody oN at 4 °C with constant rotation. After incubation with the secondary antibody, either Pierce™ ECL Western blotting Substrate or SuperSignal™ West Femto Maximum Sensitivity Substrate was added on top of the membrane. Analysis was carried out using the Chemicdoc MP Imaging System (Bio-Rad), and pictures were processed using Image Lab 5.2.1.

### 2.7. Immunofluorescence Staining

Protein overexpression and mitochondrial localization were assessed via immunofluorescence staining. Transfected cells grown on PDL-coated coverslips were fixed with 3.7% formaldehyde in PBS. After a washing step, cells were blocked and permeabilized with 5% bovine serum albumin (BSA) and 0.2% Triton X-100 in PBS for 10 min. Cells were washed and incubated with primary antibody at 4 °C. Then, cells were washed again and incubated with secondary antibody for 2 h at RT. Nuclei were stained using the NucBlue™ Live Cell reagent (Invitrogen, Waltham, MA, USA), before the coverslips were mounted on slides and left to dry for 24 h at RT. Immunofluorescence was analyzed using the Axio Imager.2M with Apotome.2 and Axiocam 705 mono (Zeiss, Oberkochen, Germany).

### 2.8. Enzyme Activity Assay

Protein functionality was verified using a colorimetric activity assay by monitoring the reduction in 2,6-dichloroindophenol (DCIP) spectrophometrically at 600 nm for 60 min. Protein concentration in the mitochondria fraction was determined using a Qubit™ Protein Assay Kit (Invitrogen) according to the manufacturer’s protocol. The activity assay was performed in a 96-well plate and reduction was measured using a Safire2 microplate reader (Tecan). Each 100 µL reaction mixture contained 5 µL of 2 mM PMS, 5 µL of 1 mM DCIP, 10 µL of 1.5 mM L2HG, and 20 µg of mitochondrial protein in HEPES-based buffer. Absorbance at 600 nm was measured every minute over 60 min using a microplate reader at RT. For each condition, a substrate-free control (without L2HG) and a blank control (without substrate and without mitochondria) were included.

### 2.9. Statistical Analysis

Quantitative data are presented as mean ± standard deviation (SD) from at least three independent experiments. Statistical analyses were performed using GraphPad Prism version 9.0 (GraphPad Software). Comparisons between two groups were performed using unpaired two-tailed Student’s *t*-tests. A *p*-value < 0.05 was considered statistically significant.

## 3. Results

### 3.1. Clinical Presentation

**Figure 1:**
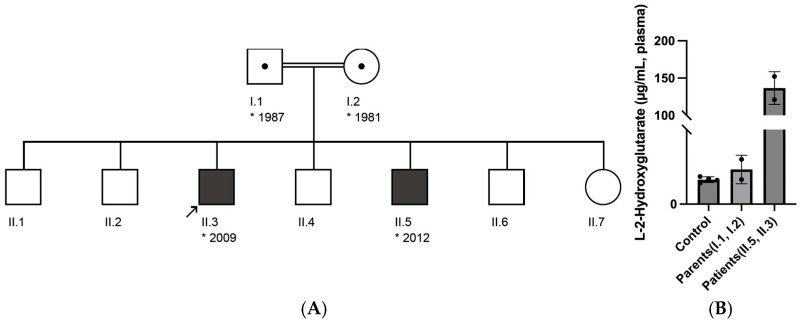
(**A**) Pedigree of the family showing two affected siblings (II.3 and II.5) with a homozygous *L2HGDH* c.905C>T p.(Pro302Leu) variant inherited from heterozygous carrier parents. (**B**) Quantification of plasma L-2-hydroxyglutarate concentrations in affected individuals, their parents, and unrelated healthy controls, measured by LC-MS. The affected siblings, both homozygous for the L2HGDH c.905C>T p.(Pro302Leu) variant, displayed significantly elevated L-2-hydroxyglutarate levels, with a group average of 136.711 µg/mL. Their heterozygous parents showed intermediate concentrations, averaging 9.302 µg/mL. In contrast, unrelated controls carrying the wild-type allele exhibited low physiological levels, with a group mean of 6.557 µg/mL.

Two male siblings, born to consanguineous parents (first cousins), presented with developmental delay and with recurrent (febrile) seizures (Figure 1A). The older brother exhibited early psychomotor stagnation, with delayed acquisition of motor milestones—crawling at approximately 4 years of age and assisted ambulation initiated between 5 and 6 years. Expressive language was markedly limited; first words were reported at age 7, though sentence formation had not been achieved. Formal cognitive assessment indicated profound intellectual disability, with estimated developmental functioning below 2 years and 3 months of age. He experienced multiple episodes of generalized tonic–clonic seizures in the context of febrile illness and was admitted to the hospital following a prolonged seizure accompanied by impaired consciousness. Neurological examination revealed generalized hypotonia, dysarthria, and an unsteady, broad-based gait. The younger sibling demonstrated a milder phenotype, characterized by global developmental delay and recurrent febrile seizures. Gross motor development was within normal limits; however, endurance was reduced, and expressive language was restricted to two-word phrases in the native language. Cranial MRI in the index case (older sibling) revealed extensive, bilateral T2-weighted hyperintensities involving the subcortical white matter with frontal predominance, as well as symmetric signal abnormalities in the basal ganglia and dentate nuclei. This radiological pattern is consistent with the characteristic imaging findings of L-2-hydroxyglutaric aciduria and supports the diagnosis of a neurometabolic leukoencephalopathy. Plasma L-2-hydroxyglutarate levels were markedly elevated in both affected siblings—152.216 µg/mL in II.5 and 121.206 µg/mL in II.3—significantly higher than those observed in their heterozygous parents and unrelated healthy controls (all < 13 µg/mL) (Figure 1B).

### 3.2. Genetic Analysis

**Figure 2:**
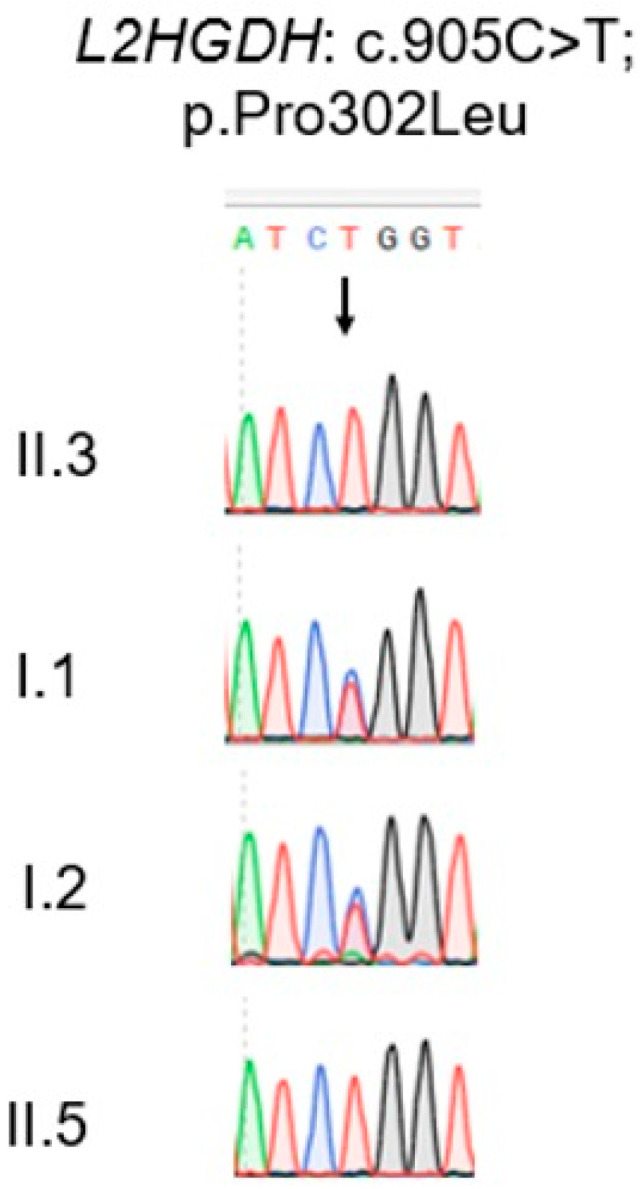
Sanger sequencing electropherogram of the *L2HGDH* gene showing the c.905C>T p.(Pro302Leu) variant in affected and carrier individuals. Sanger sequencing was performed to confirm the presence of the *L2HGDH* c.905C>T variant. The two affected individuals (II.3 and II.5) are homozygous for the T allele, showing a single peak at the mutant site. The parents (I.1 and I.2) are heterozygous carriers, as evidenced by overlapping C and T peaks at the same position. The arrow indicates the position of the nucleotide substitution.

Sanger sequencing confirmed that both affected boys are homozygous for the *L2HGDH-variant* c.905C>T, and each parent is heterozygous for the same variant (Figure 2), consistent with autosomal recessive inheritance. c.905C>T transition in exon 7 predicts a proline to leucine substitution at amino acid position 302 [p.(Pro302Leu)]. The Pro302 residue is highly conserved among *L2HGDH* orthologs, suggesting that this substitution is likely deleterious. Consistently, ClinVar lists this variant as likely pathogenic. These findings support *L2HGDH* p.(Pro302Leu) as the causal variant in this family.

### 3.3. L2HGDH Protein Expression and Processing

**Figure 3:**
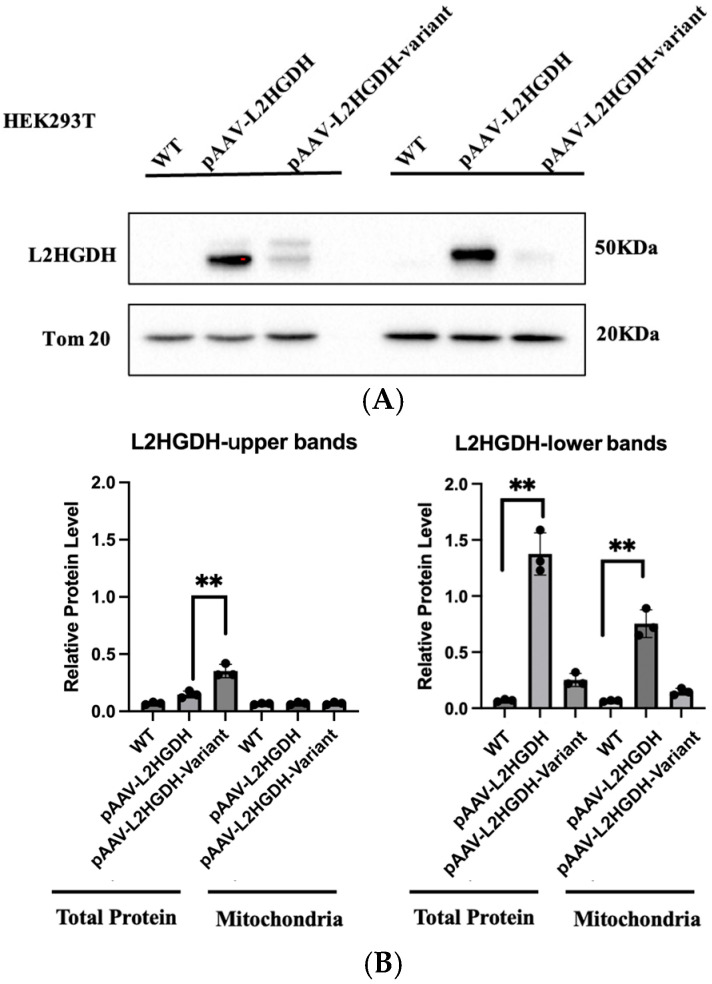

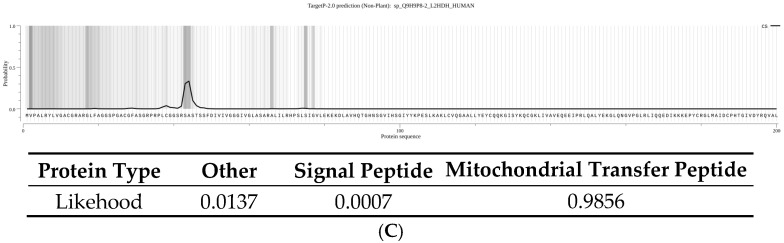
(**A**) Western blot analysis showing L2HGDH expression in total protein and mitochondrial (Mitro) fractions of HEK293 cells. “WT” indicates untransduced control cells; “pAAV-*L2HGDH*” indicates cells transduced with wild-type L2HGDH; “pAAV-*L2HGDH*-Variant” refers to cells transduced with the mutant (p.Pro302Leu) *L2HGDH*. In total protein lysates, cells transduced with wild-type L2HGDH show two bands at ~50 kDa and ~45 kDa, corresponding to the unprocessed precursor and the cleaved mature protein, respectively. In contrast, the p.Pro302Leu mutant exhibits a prominent upper ~50 kDa band than in the wild-type L2HGDH signal and a markedly reduced lower ~45 kDa band, suggesting impaired mitochondrial processing. In mitochondrial fractions, wild-type L2HGDH is strongly enriched at ~45 kDa, while mutant protein shows minimal signal, indicating defective import into mitochondria. Tom20 was used as a mitochondrial loading control. (**B**) Quantification of L2HGDH band intensity normalized to Tom20. Each bar represents the mean ± SD of three independent experiments. Individual data points are shown. ** indicates *p* < 0.01, representing statistically significant differences between groups (two-tailed unpaired *t*-test). (**C**) Prediction of mitochondrial targeting signal (MTS) in human L2HGDH using TargetP 2.0. TargetP 2.0 analysis predicts that the human L2HGDH protein (UniProt ID: Q9H9P8) contains a mitochondrial targeting peptide with high confidence. The mitochondrial transfer peptide score is 0.9856, indicating a strong likelihood of mitochondrial localization. The predicted cleavage site (CS) is located between amino acid residues 44 and 45, corresponding to an estimated molecular weight of ~4.8 kDa, which aligns with the ~5 kDa shift observed in the Western blot. The analysis was performed in non-plant mode, and the black curve in the probability plot represents the likelihood of a mitochondrial targeting signal across the N-terminal region.

To investigate the functional impact of the p.(Pro302Leu) variant, we cloned the human *L2HGDH* cDNA with a C-terminal HA tag into an AAV expression vector under the control of the CAG promoter. Both wild-type L2HGDH and the c.905C>T p.(Pro302Leu) mutant constructs were generated for downstream functional assays. These constructs were transfected into HEK 293T cells, and protein expression was assessed by Western blotting with an anti-L2HGDH antibody. In untransfected HEK293 cells, L2HGDH expression was nearly undetectable, indicating weak expression. In contrast, cells transfected with the wild-type *L2HGDH* construct exhibited two distinct bands: a faint upper band at approximately 50 kDa and a more intense lower band at around 45 kDa (Figure 3A,B). This banding pattern is consistent with previous reports [10] and is presumed to represent the unprocessed precursor and the processed mature form of the protein, respectively. Analysis using the TargetP 2.0 online prediction tool (https://services.healthtech.dtu.dk/service.php?TargetP-2.0, accessed on 16 June 2025) further supports this interpretation, indicating a 98.56% probability that L2HGDH contains a mitochondrial targeting sequence (Figure 3C). TargetP 2.0 analysis predicts the cleavage site to occur between amino acid residues 44 and 45, indicating that the mitochondrial targeting sequence (MTS) is approximately 44 amino acids in length. Given that the average molecular weight of an amino acid is ~110 Da, this corresponds to a mass of approximately 4.8 kDa. This estimation closely matches the ~5 kDa difference observed between the upper (~50 kDa) and lower (~45 kDa) bands on the Western blot, further supporting that the two bands represent the unprocessed precursor and the processed mature form of L2HGDH, respectively. In contrast, cells expressing the p.(Pro302Leu) mutant displayed a distinct band intensity pattern: the upper ~50 kDa band was more prominent than in the wild-type L2HGDH signal, while the lower ~45 kDa band accounted for approximately 10–15% of the wild-type L2HGDH signal. This suggests impaired mitochondrial import or processing efficiency of the mutant protein.

### 3.4. Enzymatic Activity Assay

**Figure 4:**
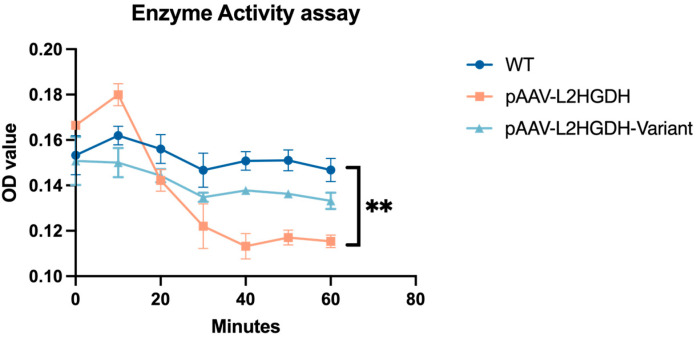
Time-course analysis of mitochondrial L2HGDH enzyme activity in untransfected, pAAV-*L2HGDH*, and pAAV-*L2HGDH*-M transfected cells showing impaired activity in mutant L2HGDH. Mitochondrial L2HGDH enzymatic activity was measured in HEK293 cells at multiple time points after transfection. Cells expressing wild-type *L2HGDH* (pAAV-*L2HGDH*) showed significantly elevated activity compared to untransfected WT and mutant (pAAV-*L2HGDH*-M) groups. In contrast, the mutant construct shows reduced enzymatic function. Statistically significant differences between groups were observed at 40, 50, and 60 min (** indicates *p* < 0.01), with no significance at other time points. Data are presented as mean ± SD (*n* = 3), and comparisons were assessed using two-way ANOVA.

Finally, to assess the functional impact of the p.(Pro302Leu) variant on L2HGDH enzymatic activity, a DCIP-based spectrophotometric assay was performed using mitochondria isolated from HEK293T cells. Three groups were included in the experiment: untransfected wild-type cells (WT), cells transfected with pAAV-*L2HGDH*, and cells transfected with the mutant construct pAAV-*L2HGDH*-Variant. As shown in Figure 4, mitochondrial lysates from the pAAV-*L2HGDH* group exhibited a clear decrease in OD600 over time, indicating strong L2HGDH activity and efficient oxidation of L-2-hydroxyglutarate. In contrast, OD values in the p.(Pro302Leu) mutant group remained largely unchanged and were comparable to those of the untransfected WT group, suggesting that both lacked significant L2HGDH activity. This is consistent with the Western blot results shown in Figure 3B. These findings indicate that the p.Pro302Leu variant compromises the catalytic function of L2HGDH, which likely accounts for the biochemical and clinical phenotype observed in the patients.

### 3.5. Subcellular Localization by Immunofluorescence

**Figure 5:**
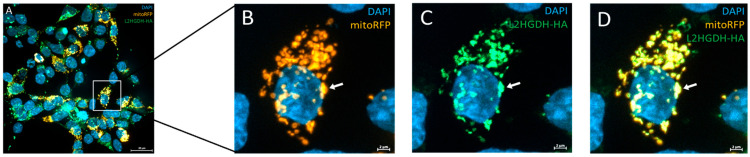
Subcellular localization of wild-type L2HGDH-HA in HEK293T cells. (**A**) Representative immunofluorescence image of HEK293T cells transiently transfected with pAAV-HA-tagged *L2HGDH*. Cells were co-stained with anti-HA antibody (green), mitoRFP (orange), and DAPI (blue). Wild-type *L2HGDH*-HA displays a punctate distribution pattern that overlaps extensively with mitochondrial signal, indicating correct mitochondrial localization. (**B**) mitoRFP (orange) and DAPI (blue); (**C**) L2HGDH-HA (green) and DAPI (blue); (**D**) Merged image showing strong colocalization (yellow) of L2HGDH-HA with mitochondria. White arrows highlight representative regions of colocalization.

**Figure 6:**
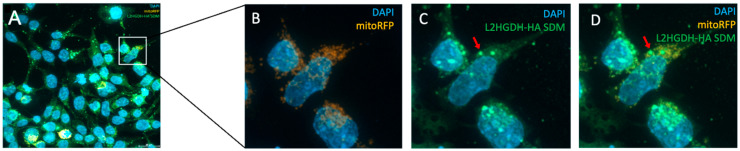
Subcellular localization of p.Pro302Leu mutant *L2HGDH* in HEK293T cells analyzed by immunofluorescence microscopy 24 h post-transfection. (**A**) Overview of transfected cells: mitochondria were labeled with mitoRFP (orange), nuclei with DAPI (blue), and mutant L2HGDH protein with anti-HA antibody (green). (**B**) Enlarged view of the boxed region in (**A**), showing the distribution of mitoRFP-labeled mitochondria and DAPI-stained nuclei. (**C**) Anti-HA staining reveals the diffuse and punctate cytoplasmic distribution of the mutant L2HGDH protein. (**D**) The merged image demonstrates minimal colocalization between the mutant protein and mitochondria. Aberrant cytoplasmic aggregates of the mutant protein are indicated by red arrows.

To investigate the subcellular localization of L2HGDH, we performed immunofluorescence staining in HEK293T cells transiently transfected with HA-tagged *L2HGDH* and p.Pro302Leu mutant *L2HGDH* constructs. Cells were stained with an anti-HA antibody to detect L2HGDH (green), mitoRFP to label mitochondria (orange), and DAPI to visualize nuclei (blue). As shown in Figure 5A–D, wild-type *L2HGDH*-HA displayed a distinct punctate staining pattern that closely overlapped with mitochondrial markers, consistent with proper mitochondrial targeting. The merged images revealed strong colocalization of green and orange signals, producing a prominent yellow signal, further confirming mitochondrial localization. In contrast, cells expressing the p.(Pro302Leu) mutant exhibited a markedly altered distribution pattern: the mutant protein appeared diffusely and was granularly distributed throughout the cytoplasm, with minimal overlap with mitochondrial markers (Figure 6A–D). Notably, aberrant cytoplasmic aggregates were frequently observed (indicated by red arrows, Figure 6D). These findings indicate that the p.Pro302Leu variant disrupts the mitochondrial targeting of L2HGDH, likely due to impaired protein folding or defects in mitochondrial import, resulting in mislocalization and cytoplasmic aggregation of the enzyme.

### 3.6. Structural Modeling and Stability Prediction

**Figure 7:**
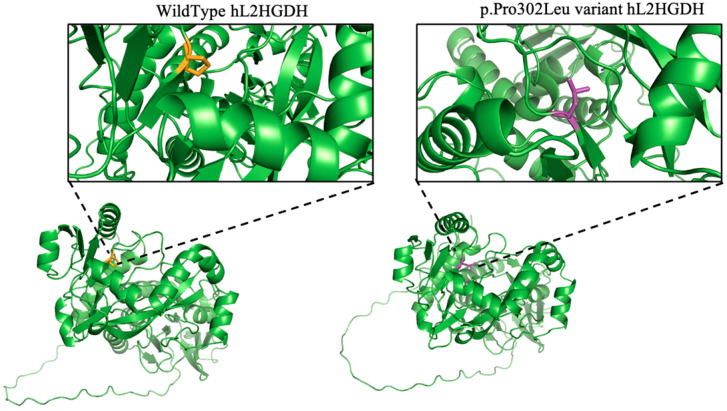
Structural modeling of wild-type (**left**) and p.Pro302Leu mutant (**right**) human L2HGDH shows an enlarged view (insets) of residue 302 and its local structural environment. Residue 302 is highlighted in orange (WT, proline) and purple (Pro302Leu mutant, leucine). Insets show the local environment of the mutated residue.

To explore the structural impact of the p.Pro302Leu variant, we employed AlphaFold to predict the full-length 3D structures of both wild-type and mutant L2HGDH proteins. Structural visualization and comparison were conducted using PyMOL. As shown in Figure 7, the variant involves a substitution of proline (Pro) at position 302 with leucine (Leu), located within a loop region forming a tight turn in the wild-type protein. This residue lies at a structural kink but is distant from the predicted active site, suggesting it does not directly interfere with substrate binding. Nonetheless, replacement of the rigid proline with a bulkier and more flexible leucine appears to relax the local loop conformation, potentially affecting folding dynamics and structural integrity.

## 4. Discussion

We have identified a homozygous *L2HGDH* variant, c.905C>T p.(Pro302Leu), in two siblings affected by L-2-hydroxyglutaric aciduria and demonstrated through functional assays that this variant disrupts the L2HGDH enzyme’s localization and activity. The clinical presentation of the two patients—developmental delay, seizures, hypotonia, and characteristic MRI white matter changes is fully consistent with the known phenotype of L2HGA. By combining clinical and molecular analyses, our study provides strong evidence that the p.(Pro302Leu) variant is pathogenic and explains the patients’ symptoms. Using a combination of differential centrifugation and Western blot, as well as immunofluorescence microscopy and a specific enzymatic assay, we postulate that this rare variant does not primarily affect enzymatic activity but impairs mitochondrial targeting and protein stability, ultimately resulting in loss of function.

Most *L2HGDH* variants reported to date cause a complete or near-complete loss of L2HGDH enzyme function [11], and p.Pro302Leu appears to follow this paradigm. The affected proline at position 302 is evolutionarily conserved, suggesting it plays an important role in the enzyme’s structure. Our results showed that the p.Pro302Leu mutant protein is present predominantly as an unprocessed precursor that fails to reach the mitochondria. Our data support that L2HGDH is a nuclear-encoded mitochondrial enzyme, synthesized in the cytosol with an N-terminal mitochondrial targeting sequence and then imported into the mitochondrial matrix or inner membrane. During import, the targeting presequence (44 amino acids) is cleaved off by mitochondrial processing peptidases, yielding the mature enzyme. In cells expressing the p.Pro302Leu mutant, we observed an accumulation of the full-length precursor form and a lack of the cleaved mature form, indicating a defect in the mitochondrial import of the mutant protein. Notably, the Pro302Leu substitution lies well beyond the N-terminal targeting signal, in the middle of the protein sequence, and therefore does not directly disrupt the signal peptide.

We propose that the p.Pro302Leu variant impairs mitochondrial import of L2HGDH by disrupting proper protein folding or structural stability. This hypothesis is supported by AlphaFold3 predicted models and structural comparison using PyMOL, which revealed a distinct local conformational alteration in the loop surrounding residue 302, despite the overall structural similarity between the wild-type and mutant proteins. Proline is among the most conformationally restricted amino acids and is frequently found at the termini of α-helices or within β-turns. In L2HGDH, Pro302 is located at the junction of a β-sheet and a loop, contributing to the stabilization of a structural turn. Substitution at this site is predicted to release backbone strain, induce local distortion, and potentially displace adjacent structural elements.

The Pro302Leu variant replaces this proline with leucine, a bulkier and more flexible hydrophobic residue. This alteration may disrupt the native turn architecture, leading to local misfolding. Although Pro302 is not situated within the predicted catalytic core (substrate-binding residues are located at positions 89, 195, and 402–404), the resulting conformational changes may compromise folding efficiency, mitochondrial recognition, or protein stability. In silico stability assessment using DynaMut2 predicted a mildly destabilizing effect, with a ΔΔG of −0.45 kcal/mol, further supporting a model in which reduced structural stability contributes to pathogenicity. Misfolded L2HGDH proteins are unlikely to be recognized by the mitochondrial import machinery, thereby failing to undergo translocation and subsequently being targeted for cytosolic degradation. Similar mitochondrial import deficiencies have been reported for other mitochondrial proteins, such as mutant COA7, which fails to enter mitochondria due to misfolding and is degraded in the cytosol [12], suggesting that misfolded L2HGDH may follow a similar fate. This interpretation is consistent with our immunoblot results, which showed that the Pro302Leu variant accumulates predominantly in its precursor form and is present at reduced steady-state levels compared to the wild-type protein. Collectively, these findings suggest that the pathogenicity of the p.Pro302Leu variant arises primarily from folding defects and import failure, rather than direct impairment of catalytic activity.

From a clinical perspective, our report underscores the value of combining genetic diagnosis with functional studies in rare metabolic disorders. The identification of L2HGDH p.Pro302Leu in these patients enabled precise genetic counseling for the family and opened the possibility of prenatal testing in future pregnancies. Notably, a ~50% reduction in enzyme activity, as may occur in heterozygous carriers such as the parents or siblings, does not lead to a clinical phenotype, suggesting that the mutant protein does not exert a dominant-negative effect or aggregate with wild-type protein in vivo.

At present, there is no definitive cure for L2HGA. Current management is primarily supportive and includes antiepileptic medication, L-carnitine supplementation (recommended dose: 50–100 mg/kg/day), physical therapy, rehabilitation, and specialized educational interventions. Only a few case reports [13,14,15] have suggested potential clinical benefits from riboflavin alone or in combination with L-carnitine. However, systematic clinical evidence remains lacking. In the present study, the p.Pro302Leu variant identified in our patients resulted in (partial) loss of L2HGDH enzymatic activity, which is likely due to impaired mitochondrial import and impaired protein stability rather than directly affecting catalysis. This raises uncertainty regarding the therapeutic efficacy of conventional metabolic supplementation in the context of such variants. Given the observed mislocalization and presumed misfolding of the mutant protein, therapeutic strategies based on molecular chaperones may warrant further investigation. For instance, the cystic fibrosis drug Trikafta [16], which combines two correctors to promote proper folding and trafficking of the F508del CFTR mutant and a potentiator to enhance channel activity, has significantly improved outcomes in patients with folding-defective variants. Whether similar strategies could be applicable to misfolded *L2HGDH* variants remains to be explored. In addition, gene therapy represents a promising future avenue for L2HGA and may offer a viable treatment option as the field advances.

## 5. Conclusions

In summary, we have characterized a *L2HGDH* missense variant, p.Pro302Leu, in two siblings with L2HGA. This variant causes the L2HGDH enzyme to mislocalize to the cytosol and lose its activity, thereby leading to the accumulation of L-2-hydroxyglutarate and the clinical manifestations of L2HGA. Our findings expand the variant spectrum of L2HGA and reveal a mechanism of pathogenicity—impaired mitochondrial import and decreased protein stability—for this missense variant. Elucidating such mechanisms is crucial for understanding the disease pathology and could inform future development of targeted treatments for this rare but debilitating neurometabolic disorder.

## Data Availability

Data are available from the corresponding author upon reasonable request.
